# What is the impact of granulocyte colony-stimulating factor (G-CSF) in subcutaneous injection or intrauterine infusion and during both the fresh and frozen embryo transfer cycles on recurrent implantation failure: a systematic review and meta-analysis?

**DOI:** 10.1186/s12958-021-00810-4

**Published:** 2021-08-13

**Authors:** Zhijin Hou, Fangjie Jiang, Jie Yang, Yang Liu, Hao Zha, Xiaoling Yang, Jia Bie, Yushi Meng

**Affiliations:** Department of Reproductive Medicine, The Second Affiliated Hospital of Kunming Medical University, NO.374 Dianmian Road, Kunming, 650101 Yunnan Province China

**Keywords:** Granulocyte colony-stimulating factor, Repeated implantation failure, Intrauterine infusion, Subcutaneous injection, Fresh embryo transfer, Frozen embryo transfer, Clinical pregnancy rate, meta-analysis

## Abstract

**Background:**

Among recurrent implantation failure (RIF) patients, the rate of successful implantation remains relatively low due to the complex etiology of the condition, including maternal, embryo and immune factors. Effective treatments are urgently needed to improve the outcomes of embryo transfer for RIF patients. In recent years, many researchers have focused on immunotherapy using granulocyte colony-stimulating factor (G-CSF) to regulate the immune environment. However, the study of the G-CSF for RIF patients has reached conflicting conclusions. The aim of this systematic review and meta-analysis was performed to further explore the effects of G-CSF according to embryo transfer cycle (fresh or frozen) and administration route (subcutaneous injection or intrauterine infusion) among RIF patients.

**Method:**

The PubMed, Embase and Cochrane Central Register of Controlled Trials (CENTRAL) databases were searched for literature published from the initial to October 2020. The meta-analysis, random-effects model and heterogeneity of the studies with I^2^ index were analyzed. Stata 15 was used for statistical analysis.

**Results:**

A total of 684 studies were obtained through the databases mentioned above. Nine RCTs included 976 RIF patients were enrolled in this meta-analysis. Subgroup analysis indicated that G-CSF improved the clinical pregnancy rate for both the fresh and frozen embryo transfer cycles (fresh RR: 1.74, 95% CI: 1.27–2.37, I^2^ = 0.0%, *n* = 410; frozen RR: 1.44, 95% CI: 1.14–1.81, I^2^ = 0.0.%, *n* = 366), and for both subcutaneous injection and intrauterine infusion (subcutaneous RR: 1.73, 95% CI: 1.33–2.23, I^2^ = 0.0%, *n* = 497; intrauterine RR: 1.39, 95% CI: 1.09–1.78, I^2^ = 0.0%, *n* = 479), but the biochemical pregnancy rate of the RIF group was also higher than that of the control group (RR: 1.85, 95% CI: 1.28–2.68; I^2^ = 20.1%, *n* = 469). There were no significant differences in the miscarriage rate (RR: 1.13, 95% CI: 0.25–5.21: I^2^ = 63.2%, *n* = 472) and live birth rate (RR: 1.43, 95% CI: 0.86–2.36; I^2^ = 52.5%; *n* = 372) when a random-effects model was employed.

**Conclusion:**

The administration of G-CSF via either subcutaneous injection or intrauterine infusion and during both the fresh and frozen embryo transfer cycles for RIF patients can improve the clinical pregnancy rate. However, whether G-CSF is effective in improving livebirth rates of RIF patients is still uncertain, continued research on the utilization and effectiveness of G-CSF is recommended before G-CSF can be considered mainstream treatment for RIF patients.

## Background

After more than 4 decades of development, assisted reproductive technology (ART) has become one of the main ways to treat infertility. However, recurrent implantation failure has consistently been a bottleneck preventing infertile patients from achieving a better pregnancy during in vitro fertilization and embryo transplantation (IVF-ET). RIF usually refers to a history of embryo transplantation failure following several ART-based procedures. A positive blood HCG test indicates the initial implantation of the embryo [1], and the absence of an intrauterine gestational sac on ultrasound examination is defined as embryo implantation failure [2]. An international consensus on the definition of embryo implantation failure has yet to be reached, however. Many researchers consider RIF patients to be women under the age of 40 years who have failed to achieve a clinical pregnancy after the transfer of at least four good-quality embryos in a minimum of three IVF fresh or frozen cycles [3]. One cause of RIF is maternal factors, such as immune rejection and problems with endometrial receptivity and the intrauterine environment. Another cause of RIF is embryonic factors, such as poor developmental potential and chromosome aneuploidy [4]. Many treatments for these problems have been found, such as hysteroscopy and preimplantation genetic diagnosis (PGT). However, the way to address the immune problem remains unclear. In recent years, some research has focused on improving endometrial receptivity by using G-CSF to address the self-immune problem.

Granulocyte colony stimulating factor (G-CSF) is a glycoprotein secreted by endothelial cells, macrophages and other immune cells that functions as a growth factor and cytokine. The functional sites of G-CSF are widespread in the human body; some studies have reported [5–7] that the receptor for G-CSF can be found not only on stromal cells, endothelial cells, bone marrow cells, fibroblasts, monocytes and macrophages but also on cells in the reproductive system, such as placental cytotrophoblasts, syncytiotrophoblasts, decidual stromal cells, endometrial glandular cells and follicular cells. G-CSF is known for its specific effects on the activation of intracellular signaling pathways that are associated with cell proliferation and differentiation and was first used for patients with myelosuppression and severe neutropenia [8, 9]. Recently, researchers found that G-CSF induces trophoblast proliferation, invasion and maintenance during pregnancy. Other researchers found that it improved endometrial receptivity for patients with RIF by promoting endometrial vascular remodeling, embryo adhesion and invasion and regulating endometrial immunity. It can also inhibit apoptosis to maintain endometrial growth [10]. G-CSF also plays an essential role in embryo implantation by regulating the expression of genes associated with embryo adhesion, cell migration, tissue remodeling and angiogenesis, which are essential for endometrial growth, successful embryo implantation and further formation of the placenta [11]. In addition, G-CSF might be involved in the induction of adaptive changes that favor immune tolerance in pregnancy; due to the semi allogenic nature of the fetus, pregnancy represents an immune challenge to the mother. G-CSF switches the T-cell cytokine secretion profile to Th2 responses and promotes IL-10-producing regulatory T-cell and tolerogenic dendritic cell differentiation [12], which are important parts of the immunoregulatory events during the implantation period [6].

The first use of G-CSF in ART was reported by Gleicher et al. [13], who revealed that four patients with thin endometrium achieved clinical pregnancy after G-CSF treatment in their study. Following verification of the safety of G-CSF administration in infertile patients [10], an increasing number of researchers began to explore the efficacy of G-CSF on embryo transfer outcomes for RIF patients; however, the conclusions among those studies are inconsistent. Many RCTs report an improvement in the clinical pregnancy rate of RIF patients by using G-CSF over that of the control group [14–16]. However, neither the clinical pregnancy rate nor the live birth rate increased in an RCT including 157 RIF patients reported by Kalem [17]. Recently, in a meta-analysis that included 1253 infertile women, Kamath et al. reported no difference between the G-CSF group and the control group [18]. However, their target population was all infertile women undergoing ART instead of only RIF patients. Another outstanding issue is the kind of embryo transfer cycle that better fits G-CSF treatment. Some studies treated RIF patients with G-CSF in the fresh embryo transfer cycle, while others treated them in the frozen cycle. Furthermore, the best administration route for G-CSF remains uncertain. In this systematic review and meta-analysis, in addition to the clinical pregnancy rate, biochemical pregnancy rate, miscarriage rate and live birth rate, subgroup analysis was conducted on patients treated during different transplantation cycles (fresh and frozen) and with administration routes (subcutaneous injection and intrauterine perfusion) to obtain further information on the influence of G-CSF on RIF patients.

## Methods

The PubMed, Embase and Cochrane Central Register of Controlled Trials (CENTRAL) databases were searched for literature published before October 2020. The protocol of this systematic review was registered with INPLASY (registration number INPLASY202170040). The obtained studies were screened in accordance with the PICOS rule (participants, intervention, control, outcome and study type). Participants: Patients with RIF; intervention group: G-CSF; control group: placebo or no treatment; main outcome: clinical pregnancy rate; study type: RCT. The search strategy for PubMed is as follows: ((((((recurrent implantation failure [Title/Abstract]) OR (repeated implantation failure [Title/Abstract])) OR (mif [Title/Abstract])) OR (rif [Title/Abstract])) OR (((repeated [Title/Abstract]) AND (implantation [Title/Abstract])) AND (failure [Title/Abstract]))) OR (((multiple [Title/Abstract]) AND (implantation [Title/Abstract])) AND (failure [Title/Abstract]))) AND ((((((((Granulocyte Colony-Stimulating Factor [Mesh]) OR (G-CSF [Title/Abstract])) OR (Granulocyte colony stimulating factor [Title/Abstract])) OR (Granulocyte-colony stimulating factor [Title/Abstract])) OR (colony-stimulating factor [Title/Abstract])) OR (CSF [Title/Abstract])) OR (Lenograstim [Title/Abstract])) OR (Filgrastim [Title/Abstract])).

### Selection of articles

A study was included when it met all of the following criteria: 1. Patients under the age of 40 years who failed to achieve clinical pregnancy after the transfer of at least four good-quality embryos in a minimum of two IVF fresh or frozen cycles. 2. Subcutaneous injection or intrauterine infusion of G-CSF before a fresh or frozen embryo transfer cycle. 3. RCT with a control group that received placebo or no treatment. 4. Reporting of one of the following outcomes: clinical pregnancy rate, live birth rate, miscarriage rate, biochemical pregnancy rate. 5. Patients in RCT with uterine factors such as uterine anomalies, myoma, endometrial polyps were excluded. 6. Patients in RCT with systemic diseases and contraindications of G-CSF were excluded. Studies that did not meet all the criteria above were excluded.

Data extraction was performed by two review authors (Hou and Jiang) independently, and disagreement was resolved by discussion or decided by a third review author (Meng). Randomized controlled trials were assessed for risk of bias using the Cochrane ‘Risk of bias’ tool according to the criteria outlined in the Cochrane Handbook for Systematic Reviews of Interventions (Version 5.1.0). The following domains were assessed by two researchers (Hou and Jiang) independently:

1.Selection bias (random sequence generation and allocation concealment);2. Performance bias (blinding of participants and personnel);3. Detection bias (blinding of outcome assessors);4. Attrition bias (incomplete outcome data);5. Reporting bias (selective reporting);6. Other bias (including unplanned interim analysis).

The evaluations were categorized as having ‘low risk’, ‘unclear risk’ or ‘high risk’ of bias; a detailed list is shown in Table 1.

**Table 1:** Risk of bias assessment. *①Random sequence generation (selection bias); ②Allocation concealment (selection bias); ③Blinding of participants and personal (performance bias); ④Blinding of outcome assessment (detection bias); ⑤Incomplete outcome data (attrition bias);⑥Selection reporting (reporting bias); ⑦Other bias

Study	①*	②*	③*	④*	⑤*	⑥*	⑦*
**Kalem (2020)**	Low risk	Low risk	Low risk	Low risk	Low risk	Low risk	Low risk
**Huang (2020)**	Low risk	Low risk	Unclear risk	Low risk	Unclear risk	Low risk	Unclear risk
**Scarpellini (2019)**	Low risk	Low risk	Low risk	Low risk	Low risk	Low risk	Unclear risk
**Arefi** **(2018)**	Low risk	Low risk	Low risk	Low risk	Low risk	Low risk	Low risk
**Tanha (2016)**	Low risk	Low risk	Low risk	Low risk	Low risk	Low risk	Unclear risk
**Aleyasin (2016)**	Low risk	Low risk	Low risk	Low risk	Low risk	Low risk	Low risk
**Obidniak (2016)**	Low risk	Low risk	Low risk	Low risk	Low risk	Low risk	Low risk
**Maryam (2016)**	Low risk	Low risk	Unclear risk	Low risk	Unclear risk	Low risk	Unclear risk
**Abedi (2015)**	Low risk	Low risk	Low risk	Low risk	Low risk	Low risk	Low risk

### Data analysis

Stata software (Version 15.0; Stata Corporation, College Station, TX, USA) was employed to perform all data analysis. The influence of G-CSF treatment on the outcomes of IVF-ET for RIF patients was assessed with pooled risk ratios (RRs) and their 95% confidence intervals (CIs). The pooled RRs were calculated through a Mantel–Haenszel fixed-effects model if there was no heterogeneity; otherwise, a random-effects model was adopted. Statistical heterogeneity across studies was formally tested using Cochran’s Q test. The I^2^ statistic was examined, and I^2^ > 50% was considered to represent significant heterogeneity between studies. Subgroup analysis was performed in terms of embryo transplantation cycle and administration route. We did not assess publication bias across studies since the number of included studies was under 10.

## Results

### Study selection

**Fig. 1:**
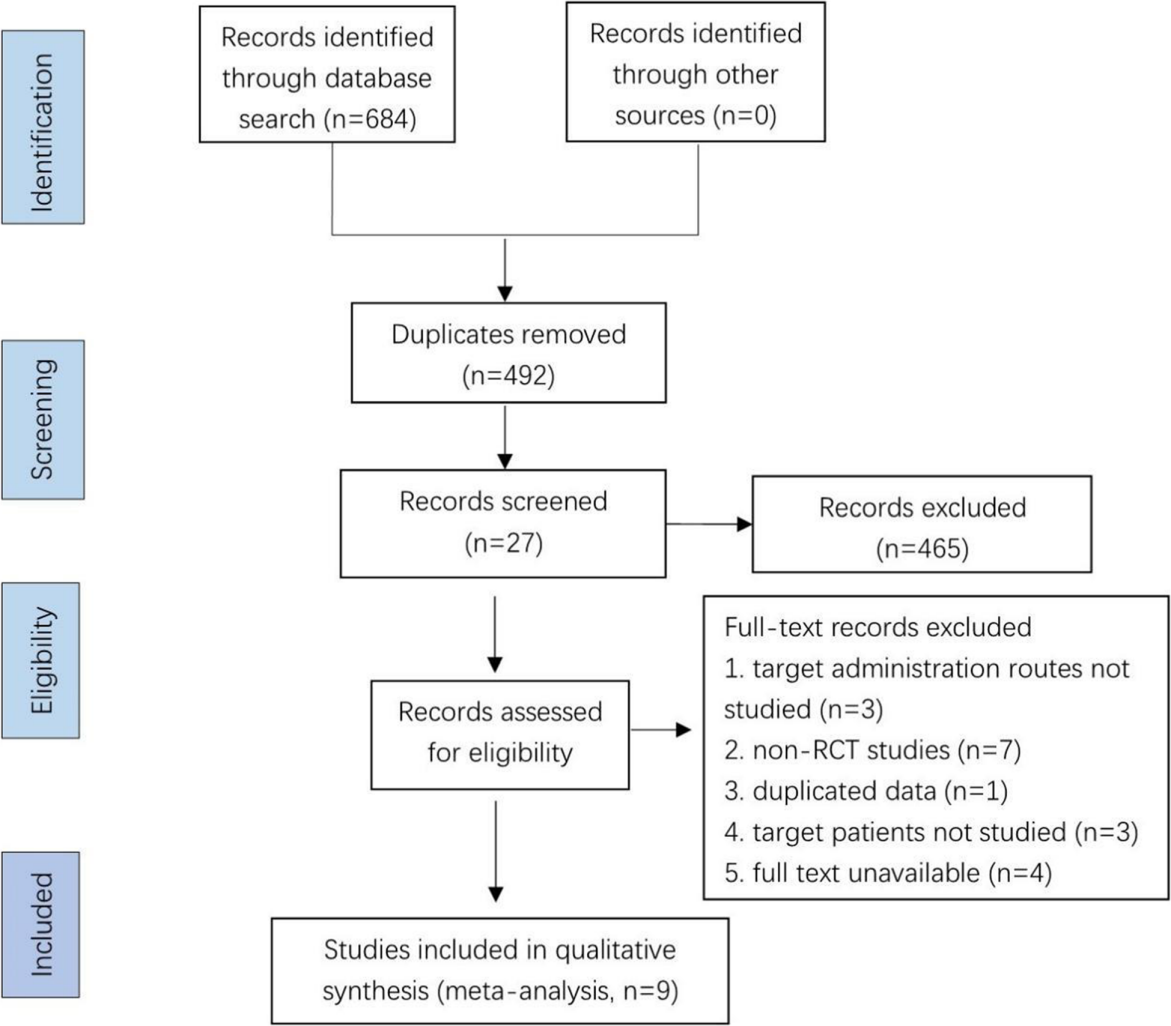
PRISMA flow diagram for identifying and selecting studies

A total of 684 studies were obtained by primary search: 230 from PubMed, 393 from Embase, and 61 from CENTRAL. After a thorough screening, 10 RCTs [15–17, 19–24] published between 2015 and 2020 were finally included. The specific steps of the study selection are shown in Fig. 1. A total of 976 patients with RIF were included, including 462 in the study group with G-CSF treatment and 524 in the control group with placebo or no treatment. There were four valid outcome indicators in the extracted data, including the clinical pregnancy rate, live birth rate, biochemical pregnancy rate, and miscarriage rate. Basic characteristics of all included studies were extracted, including author, year of publication, country of study, study period, administration route, transplantation cycle, method of intervention, definition of RIF and outcome indicators. Details of the extracted characteristics are shown in Table 2.

**Table 2:** Basic characteristic table

Author\Year \Country	study design	Study period	ET cycle	Intervention group	Control group	Definition of RIF	Outcomes
**Kalem** 2020 Turkey	RCT	2016.3–2017.12	Fresh cycle	Administered 30 mIU/ml of Leucotomy®(Filgrastim [G-CSF]; DEM Medical, Dong-A; South Korea) through slow infusion into the endometrial cavity using a soft embryo transfer catheter	1 mL normal saline of infused into the endometrial cavity in the same way as the intervention group	Failure to achieve a clinical pregnancy after the transfer of at least four good-quality embryos in a minimum of three fresh or frozen cycles to a woman under the age of 40 years	Endometrial thickness; clinical pregnancy rate. Live birth rate
**Huang** 2020 China	RCT	2015.12–2017.7	Frozen cycle	Administered a 1-ml uterine infusion of recombinant human G-CSF (150 mg, 1 ml, Rubbia, Shandong) through an intrauterine insemination (IUI) catheter	1. *n* = 52 intrauterine infusion of saline solution in the same way as the intervention group; 2. *n* = 59 no treatment	At least two previous implantation failures	Clinical pregnancy rate miscarriage rate; live birth rate
**Scarpellini** **2019** **Italy**	RCT	Not reported	Frozen cycle	Subcutaneous GM-CSF 1.5 mg/kg/daily (60–100) from the day of embryo transfer to the day of b-hcg HCG	Subcutaneous saline solution infusion in the same way as the intervention group	At least 9 good embryos previously transferred, women less than 38 years old	Pregnancy rate
**Arefi** 2018 Iran	RCT	2010.5–2015.10	Fresh cycle	Recombinant human G-CSF 300 μg (0.5 ml) subcutaneously injected 30 min before blastocyst embryo transfer	Not reported	More than three previous IVF-ET failures	Live birth rate; clinical pregnancy rate
**Tanha** 2016 Iran	RCT	2011.12–2014.1	Frozen cycle (*n* = 9) Fresh cycle(*n* = 91)	G-CSF 300 m0mg/1 m1ml was administered at the day of oocyte puncture or day of progesterone administration of FET cycle	40 in the saline group;20 in the placebo group	At least three implantation failures with history of transferring at least four good-quality embryos without uterine or thrombophilia factors	Pregnancy rate; implantation rate
**Aleyasin 2016** **Iran**	RCT	Not reported	Frozen cycle	Subcutaneous 300 μg GCSF before implantation	Not reported	Not reported	Implantation rate; chemical pregnancy rate; clinical pregnancy rate
**Obidniak** 2016 Iran	RCT	Not reported	Frozen cycle	1. Study group No 1: intrauterine perfusion with G-CSF (filgrastim 30 million IU, 1 mL) using insemination catheter 5 days prior to embryo transfer ;2. Study group No 2: G-CSF (filgrastim 30 million IU, 1 ml) was administered subcutaneously once at on the day of embryo transfer	No therapy	At least two cycles of in vitro fertilization in which good-quality embryos (Gardner blastocyst grading system) were transferred in each cycle without achieving a clinical pregnancy	Implantation rate; clinical pregnancy rate
**Maryam** 2016 Iran	RCT	2014.10–2015.2	fresh cycle	G-CSF 0.5 ml (300 μg/ml) GCSF was infused intrauterine infusion in intervention group	Not reported	Women between 20 and 40 years old with history of at least two implantation failures	Implantation rate; clinical pregnancy rate
**Abedi** 2015 Iran	RCT	Not reported	Not reported	Subcutaneous 300 m0mg GCSF before implantation	Not reported	Infertile women with normal endometrial thickness who had 2 implantation failures after IVF cycles	Implantation rate chemical pregnancy rate; clinical pregnancy rate

### Clinical pregnancy rate

**Fig. 2:**
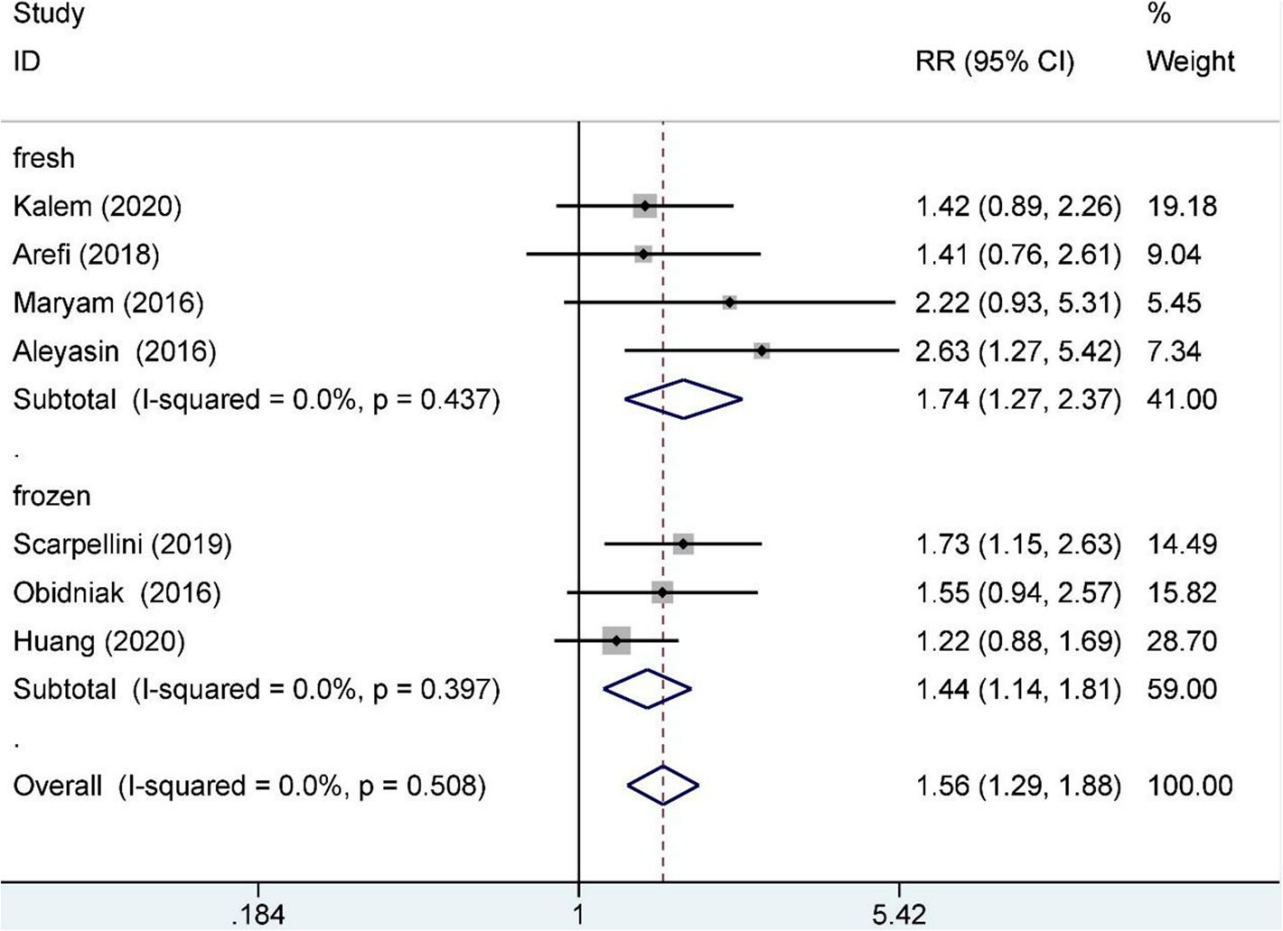
Clinical pregnancy rate subgroup analysis (transfer cycle)

**Fig. 3:**
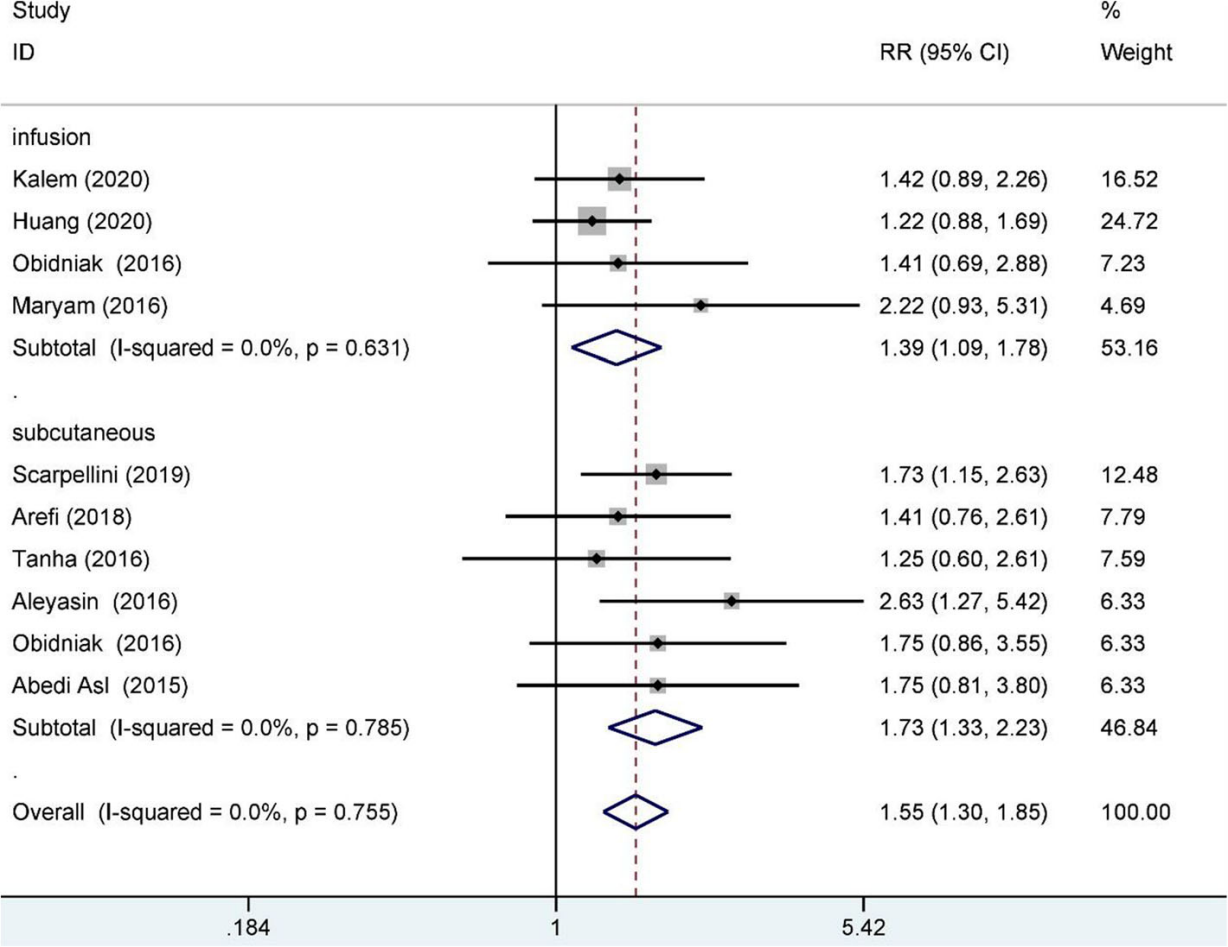
Clinical pregnancy rate subgroup analysis (route of administration)

Nine studies reported the clinical pregnancy rate and included 976 RIF patients, 462 of whom were in the study group and 524 of whom were in the control group. A fixed-effects model was used for data synthesis, and the result indicated that G-CSF treatment improved the clinical pregnancy rate for RIF patients (RR:1.55, 95% CI:1.30–1.85; I^2^ = 0.0%, *n* = 976). When subgrouped by embryo transfer cycle (410 in the fresh cycle, 366 in the frozen cycle), the result indicated that G-CSF treatment improved the clinical pregnancy rate for RIF patients for both cycles (fresh RR: 1.74, 95% CI: 1.27–2.37, I^2^ = 0.0%, *n* = 410; frozen RR: 1.44, 95% CI: 1.14–1.81, I^2^ = 0.0.%, *n* = 366); see Fig. 2 for details. When subgrouped by administration route (497 with subcutaneous injection and 479 with intrauterine infusion), the results indicated that G-CSF treatment improved the clinical pregnancy rate for RIF patients for both routes (subcutaneous RR: 1.73, 95% CI: 1.33–2.23, I^2^ = 0.0%, *n* = 497; intrauterine RR: 1.39, 95% CI: 1.09–1.78, I^2^ = 0.0%); see Fig. 3 for details.

### Biochemical pregnancy rate

**Fig. 4:**
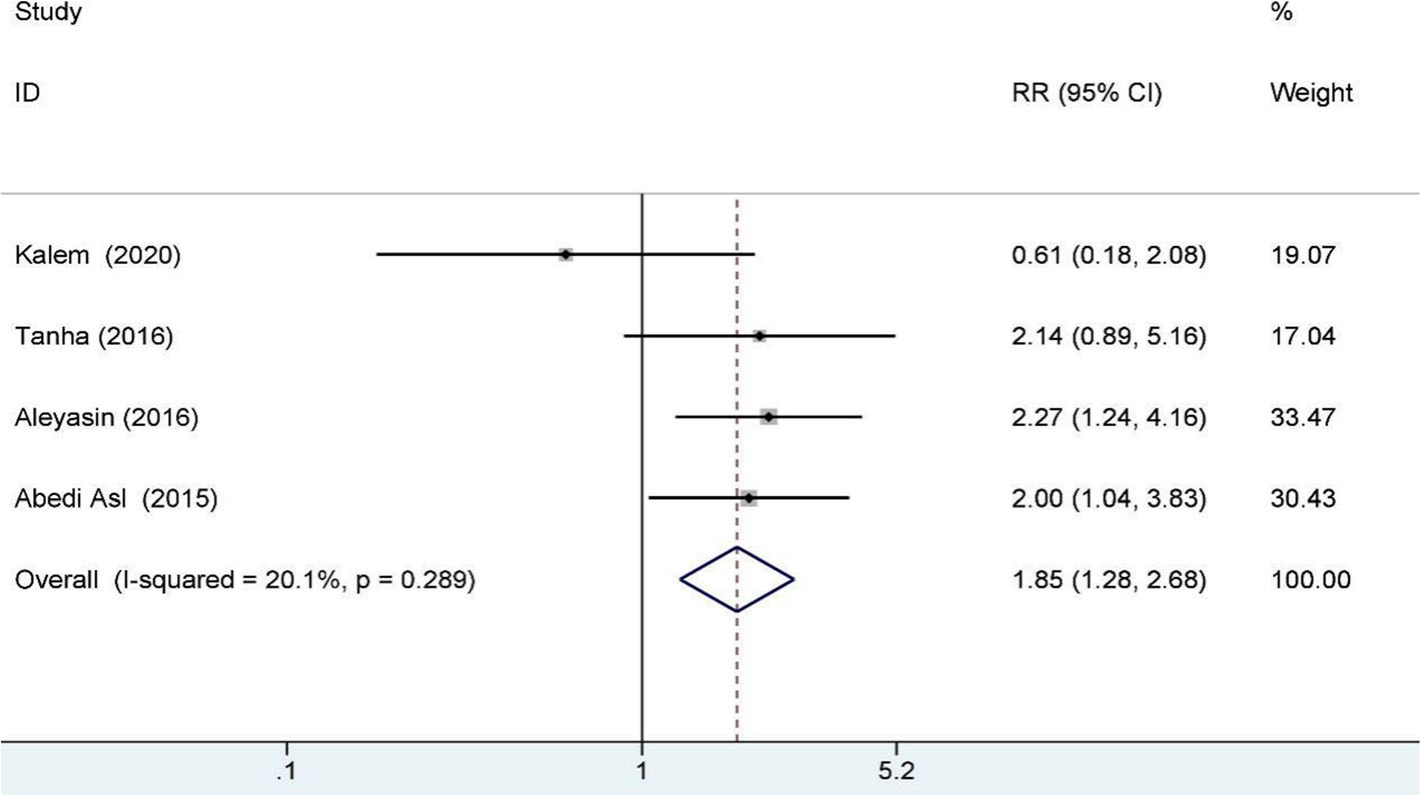
Biochemical pregnancy rate

Four studies reported the biochemical pregnancy rate and included 469 RIF patients, 228 of whom were in the study group and 241 of whom were in the control group. A fixed-effects model was used for data synthesis, and the results indicated that G-CSF treatment improved the biochemical pregnancy rate for RIF patients (RR: 1.85, 95% CI: 1.28–2.68; I^2^ = 20.1%); see Fig. 4 for details.

### Miscarriage rate

**Fig. 5:**
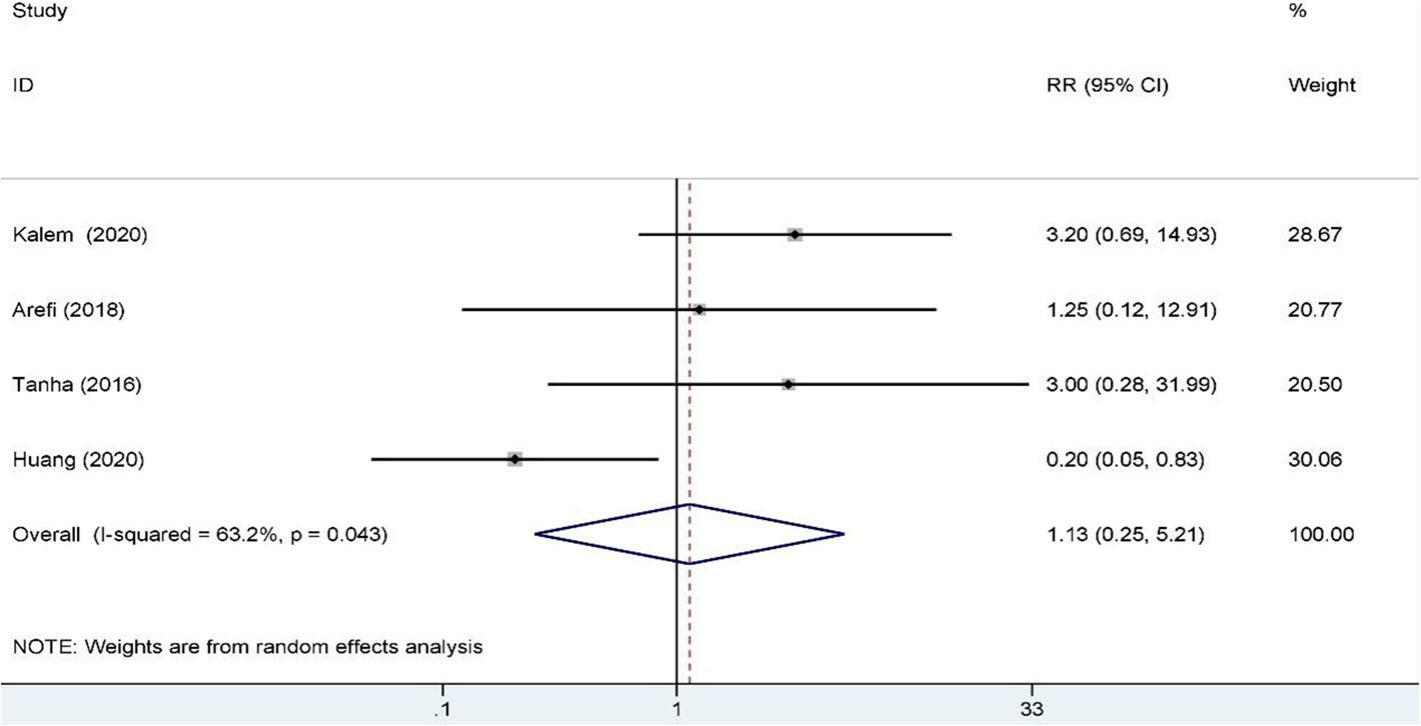
miscarriage rate

Four studies reported miscarriage rate and included 472 RIF patients, 206 of whom were in the study group and 266 of whom were in the control group. A random-effects model was used for data synthesis, and no significant difference was found between the study and control groups (RR: 1.13, 95% CI: 0.25–5.21; I2 = 63.2%); see Fig. 5 for details. An I2 of 63.2% was observed in this synthesis, indicating considerable heterogeneity, which might have been caused by the small number of included patients.

### Live birth rate

**Fig. 6:**
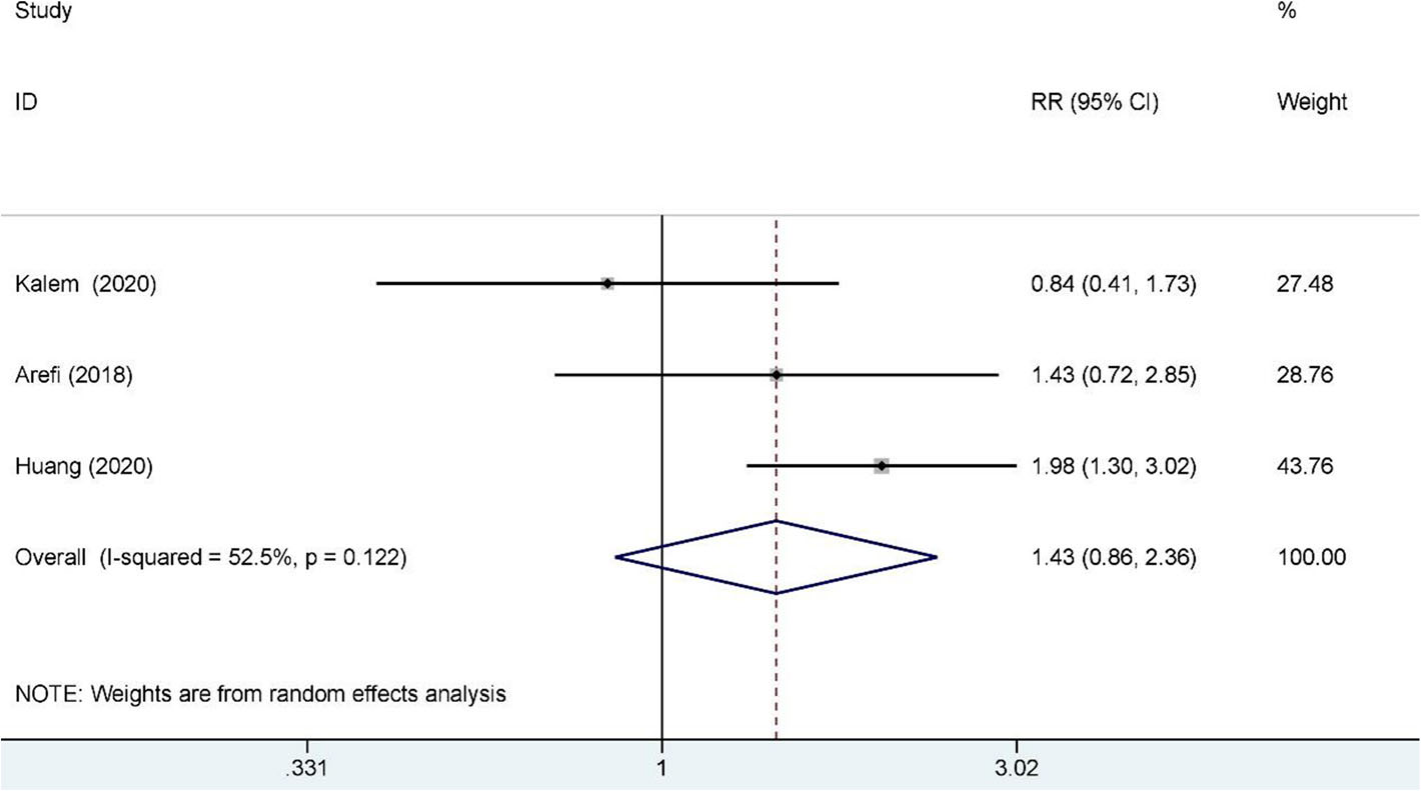
Live birth rate

Three studies reported the live birth rate and included 372 RIF patients, 166 of whom were in the study group and 206 of whom were in the control group. A random-effects model was used for data synthesis, and no significant difference was found between the study and control groups (RR: 1.43, 95% CI: 0.86–2.36; I^2^ = 52.5%); see Fig. 6 for details. An I^2^ of 52.5% was observed in this synthesis, indicating considerable heterogeneity, which might have been caused by the small number of included patients.

## Discussion

In addition to the proliferation and differentiation of granulocytes, G-CSF has a clear immunomodulatory effect. G-CSF receptors can be found throughout the reproductive system, especially in placental cytotrophoblasts, syncytiotrophoblasts, decidual stromal cells, endometrial glandular cells and follicular cells, which provide the foundation for the establishment and maintenance of pregnancy. Most studies so far have reported an improvement in the clinical pregnancy rate of RIF patients by G-CSF treatment, which coincides with our results. The reasons for this improvement may include induction of local immune regulation of the endometrium, embryo adhesion and implantation, proliferation of trophoblasts and endometrial vascular remodeling by G-CSF. As we mentioned above, the using of G-CSF improved clinical pregnancy rate of RIF patients, other studies proved that G-CSF may play an essential role before and after the establishment of pregnancy. However, few studies have compared the effect of G-CSF on different embryo transfer cycles.

Both fresh and frozen embryo transfer cycles are widely used in IVF-ET. However, the endometrial environment is not identical between these two cycles. Previous studies found that 15% of women received autoantibodies after controlled ovarian hyperstimulation (COH), which can lead to reproductive disorders such as endometriosis, recurrent abortion, and premature ovarian failure [25]. It is known that G-CSF might be involved in the induction of adaptive changes that favor immune tolerance in pregnancy, which might be an important part of the immunoregulatory events during the implantation period. In this review and meta-analysis, we performed a subgroup analysis in terms of whether the fresh or frozen cycle was used to compare the effect of G-CSF as an immune factor on patients with RIF between the two subgroups. The results indicate an increase in the pregnancy rates for both cycles. It suggested that G-CSF can be used in the fresh or frozen cycle without distinction. However, more high-quality studies with larger populations are still needed to form clinical guidelines.

Biochemical pregnancy tends to occur when the endometrial environment cannot maintain the continuation of the established pregnancy. Many previous studies [16, 23] reported an increased biochemical pregnancy rate of RIF patients after G-CSF treatment, which coincides with our results. These authors suggest that this increase in the biochemical pregnancy rate might be related to a decrease in the sustaining effect of G-CSF. G-CSF was administered at a single dose before embryo transfer in most studies included in this review and meta-analysis. This protein improves endometrial receptivity and thus the embryo implantation rate. However, with the decrease in the sustaining effects of G-CSF, some RIF patients with poor endometrial receptivity are unable to maintain pregnancy, leading to the occurrence of biochemical pregnancy. If this is true, does continuous administration of G-CSF help RIF patients maintain their hard-won pregnancy? More studies are needed to address this hypothesis.

The routine G-CSF administration routes in ART include subcutaneous injection and intrauterine infusion. However, whether one route is better than the other remains obscure. Zeyneloglu et al. reported a better embryo transfer outcome by combining subcutaneous and intrauterine G-CSF treatment for RIF patients than by subcutaneous injection alone [14]. In this review and meta-analysis, we performed a subgroup analysis by subcutaneous injection and intrauterine infusion to compare the influence of G-CSF on RIF patients. The result indicates an increase in the pregnancy rates for both routes. The G-CSF receptor can be found throughout the human body, and the systematic use of G-CSF assuredly induces more side effects than its topical use. Whether these side effects harm the pregnancy is unknown. Intrauterine infusion of G-CSF will have a direct effect on the endometrium, which might be easier and safer for the patient.

As mentioned above, G-CSF might be beneficial for the establishment and maintenance of pregnancy. The results of this review and meta-analysis show no significant effect of G-CSF treatment on the miscarriage or live birth rate. Due to the limited number of included patients, the exact effect of G-CSF on these rates. Miscarriage for RIF patients remains unclear, and more high-quality, larger and multicenter RCTs are needed to provide more authoritative evidence for clinical practice.

## Conclusion

In this review and meta-analysis, the results of data synthesis for 976 RIF patients indicate that G-CSF treatment improved the clinical pregnancy rate; however, the biochemical pregnancy rate increased as well, which is coincident with previous studies. In addition, subgroup analysis indicated that the administration of G-CSF by either subcutaneous injection or intrauterine infusion during both fresh and frozen embryo transfer cycles for RIF patients improved the clinical pregnancy rate. The influence of G-CSF on the miscarriage and live birth rates of RIF patients is unclear, and more studies are needed before G-CSF become mainstream treatment for RIF patients.

## Data Availability

The current study was based on results of relevant published studies.

## Abbreviations

G-CSF: Granulocyte colony-stimulating factor
RIF: Recurrent implantation failure
CENTRAL: Cochrane Central Register of Controlled Trials
RRs: risk Ratios
CIs: Confidence intervals
ART: Assisted reproductive technology
IVF-ET: In vitro fertilization and embryo transplantation
PGT: Preimplantation genetic diagnosis
COH: Controlled ovarian hyperstimulation

## References

[1] RPL EGGo, Bender Atik R, Christiansen OB, Elson J, Kolte AM, Lewis S, et al. ESHRE guideline: recurrent pregnancy loss. Hum Reprod Open. 2018;2018(2):hoy004. 10.1093/hropen/hoy004. 10.1093/hropen/hoy004 PMC6276652 31486805

[2] Parrella A, Keating D, Cheung S, Xie P, Stewart JD, Rosenwaks Z, Palermo GD (2019). A treatment approach for couples with disrupted sperm DNA integrity and recurrent ART failure. J Assist Reprod Genet.

[3] Shaulov T, Sierra S, Sylvestre C (2020). Recurrent implantation failure in IVF: a Canadian fertility and andrology society clinical practice G uideline. Reprod BioMed Online.

[4] Saxtorph MH, Hallager T, Persson G, Petersen KB, Eriksen JO, Larsen LG, Hviid TV, Macklon N (2020). Assessing endometrial receptivity after recurrent implantation failure: a prospective controlled coho rt study. Reprod BioMed Online.

[5] Li J, Mo S, Chen Y (2017). The effect of G-CSF on infertile women undergoing IVF treatment: a meta-analysis. Syst Biol Reprod Med.

[6] Lédée N, Lombroso R, Lombardelli L, Selva J, Dubanchet S, Chaouat G (2008). Cytokines and chemokines in follicular fluids and potential of the corresponding embryo: the role of granulocyte colony-stimulating factor. Hum Reprod.

[7] McCracken SA, Grant KE, MacKenzie IZ, Redman CW, Mardon HJ (1999). Gestational regulation of granulocyte-colony stimulating factor receptor expression in the human plac enta. Biol Reprod.

[8] Lédée N, Gridelet V, Ravet S, Jouan C, Gaspard O, Wenders F (2013). Impact of follicular G-CSF quantification on subsequent embryo transfer decisions: a proof of concept study. Hum Reprod.

[9] Saito S, Fukunaga R, Ichijo M, Nagata S (1994). Expression of granulocyte colony-stimulating factor and its receptor at the fetomaternal interface in murine and human pregnancy. Growth Factors.

[10] Kamath MS, Chittawar PB, Kirubakaran R, Mascarenhas M (2017). Use of granulocyte-colony stimulating factor in assisted reproductive technology: a systematic review and meta-analysis. Eur J Obstet Gynecol Reprod Biol.

[11] Rahmati M, Petitbarat M, Dubanchet S, Bensussan A, Chaouat G, Ledee N (2014). Granulocyte-Colony stimulating factor related pathways tested on an endometrial ex-vivo model. PLoS One.

[12] Rutella S, Zavala F, Danese S, Kared H, Leone G (2005). Granulocyte colony-stimulating factor: a novel mediator of T cell tolerance. J Immunol.

[13] Gleicher N, Vidali A, Barad DH (2011). Successful treatment of unresponsive thin endometrium. Fertil Steril.

[14] Zeyneloglu HB, Tohma YA, Onalan G, Moran U (2020). Granulocyte colony-stimulating factor for intracytoplasmic sperm injection patients with repeated imp lantation failure: which route is best??. J Obstet Gynaecol.

[15] Arefi S, Fazeli E, Esfahani M, Borhani N, Yamini N, Hosseini A (2018). Granulocyte-colony stimulating factor may improve pregnancy outcome in patients with history of unexp lained recurrent implantation failure: an RCT. Int J Reprod Biomed (Yazd).

[16] Davari-Tanha F, Shahrokh Tehraninejad E, Ghazi M, Shahraki Z (2016). The role of G-CSF in recurrent implantation failure: a randomized double blind placebo control trial. Int J Reprod Biomed (Yazd).

[17] Kalem Z, Namli Kalem M, Bakirarar B, Kent E, Makrigiannakis A, Gurgan T (2020). Intrauterine G-CSF Administration in Recurrent Implantation Failure (RIF). An Rct. Sci Rep.

[18] Kamath MS, Kirubakaran R, Sunkara SK (2020). Granulocyte-colony stimulating factor administration for subfertile women undergoing assisted reprodu ction. Cochrane Database Syst Rev.

[19] Huang P, Yao C, Wei L, Lin Z. The intrauterine perfusion of granulocyte-colony stimulating factor (G-CSF) before frozen-thawed embryo transfer in patients with two or more implantation failures. Hum Fertil. 2020:1–5. 10.1080/14647273.2020.1811904. 10.1080/14647273.2020.1811904 32862740

[20] Scarpellini F, Marco S, Stamenov G (2019). GM-CSF treatment in recurrent implantation failure women after PGS: a randomized controlled trial. Am J Reprod Immunol.

[21] Obidniak D, Gzgzyan A, Dzhemlikhanova L, Feoktistov A (2016). Effect of colony-stimulating growth factor on outcome of frozen-thawed embryo transfer in patients with repeated implantation failure. Fertil Steril.

[22] Eftekhar M, Miraj S, Farid Mojtahedi M, Neghab N (2016). Efficacy of intrauterine infusion of granulocyte colony stimulating factor on patients with history o f implantation failure: a randomized control trial. Int J Reprod Biomed (Yazd).

[23] Aleyasin A, Abediasl Z, Nazari A, Sheikh M (2016). Granulocyte colony-stimulating factor in repeated IVF failure, a randomized trial. Reproduction.

[24] Abedi AZ (2015). The efficacy of systemic administration of granulocyte colony stimulating factor (GCSF) on the in vitro fertilization (IVF) success in women with repeated implantation failure. Fertil Steril.

[25] Irvine WJ, Chan MM, Scarth L, Kolb FO, Hartog M, Bayliss RI (1968). Immunological aspects of premature ovarian failure associated with idiopathic Addison's disease. Lancet.

